# Effect sizes in ongoing randomized controlled critical care trials

**DOI:** 10.1186/s13054-017-1726-x

**Published:** 2017-06-05

**Authors:** Elliott E. Ridgeon, Rinaldo Bellomo, Scott K. Aberegg, Rob Mac Sweeney, Rachel S. Varughese, Giovanni Landoni, Paul J. Young

**Affiliations:** 1 Medical Research Institute of New Zealand, Wellington, New Zealand; 2 Australian and New Zealand Intensive Care Research Centre, School of Public Health and Preventive Medicine, Monash University, Melbourne, Australia; 3 Intensive Care Unit, Austin Hospital, Melbourne, Australia; 4 Division of Pulmonary and Critical Care Medicine, University of Utah School of Medicine, Salt Lake City, UT USA; 5 Intensive Care Unit, Royal Victoria Hospital, Belfast, UK; 6 Department of Anesthesia and Intensive Care, Istituto di Ricovero e Cura a Carattere Scientifico (IRCCS), San Raffaele Scientific Institute, Milan, Italy; 7 Vita-Salute San Raffaele University, Milan, Italy; 8 Intensive Care Unit, Wellington Regional Hospital, Wellington, New Zealand

**Keywords:** Intensive care unit, Critical care, Intensive care, Randomized clinical trial, Clinical trial design

## Abstract

**Background:**

An important limitation of many critical care trial designs is that they hypothesize large, and potentially implausible, reductions in mortality. Interpretation of trial results could be improved by systematic assessment of the plausibility of trial hypotheses; however, such assessment has not been attempted in the field of critical care medicine. The purpose of this study was to determine clinicians’ views about prior probabilities and plausible effect sizes for ongoing critical care trials where the primary endpoint is landmark mortality.

**Methods:**

We conducted a systematic review of clinical trial registries in September 2015 to identify ongoing critical care medicine trials where landmark mortality was the primary outcome, followed by a clinician survey to obtain opinions about ten large trials. Clinicians were asked to estimate the probability that each trial would demonstrate a mortality effect equal to or larger than that used in its sample size calculations.

**Results:**

Estimates provided by individual clinicians varied from 0% to 100% for most trials, with a median estimate of 15% (IQR 10–20%). The median largest absolute mortality reduction considered plausible was 4.5% (IQR 3.5–5%), compared with a median absolute mortality reduction used in sample size calculations of 5% (IQR 3.6–10%) (*P* = 0.27).

**Conclusions:**

For some of the largest ongoing critical care trials, many clinicians regard prior probabilities as low and consider that plausible effects on absolute mortality are less than 5%. Further work is needed to determine whether pooled estimates obtained by surveying clinicians are replicable and accurate or whether other methods of estimating prior probability are preferred.

**Electronic supplementary material:**

The online version of this article (doi:10.1186/s13054-017-1726-x) contains supplementary material, which is available to authorized users.

## Background

Mortality measured at a particular time point (landmark mortality) is often regarded as the gold standard outcome for randomized controlled trials in critical care medicine [1]. However, the utility of trials in generating evidence for interventions to increase survival in intensive care unit (ICU) patients has been disputed [2–4].

An important limitation of many critical care trials is that they hypothesize large and potentially implausible reductions in absolute mortality [5]. This is a major problem in trial design for two reasons. First, it makes a type II error (false-negative) more likely. Second, the less plausible a postulated mortality reduction is, the more likely it is that a statistically significant mortality difference will represent a type I error (false-positive) [6]. This is because a *P* value is defined as the probability of finding a result equal to or more extreme than that actually observed, under the assumption that the null hypothesis is true. This means that the greater the pretrial chance or prior probability that the null hypothesis is correct, the lower the chance that a *P* value below a particular significance threshold will represent a true-positive. Thus, estimating the plausibility of a trial’s hypothesis on the basis of prior knowledge has the potential to aid in the interpretation of the results [7]. However, assessment of such prior probability is problematic and rarely discussed. This is because it is likely to be subjective, to be based on limited data, and to have a wide range of possible values. Systematic reporting of clinicians’ estimates of prior probability for clinical trials has not previously been attempted in the field of critical care medicine.

Accordingly, the primary aim of this study was to develop data-driven estimates of prior probability for some of the largest ongoing trials in critically ill adults where the primary endpoint is landmark mortality. We hypothesized that surveyed clinicians’ estimates of prior probability would be consistently low and that effect sizes regarded as plausible by clinicians would be smaller than those postulated by investigators.

## Methods

### Study design

We conducted a systematic review of databases of registered clinical trials, followed by a clinician survey.

### Systematic review

The EudraCT, ClinicalTrials.gov, and ANZCTR clinical trial registries were searched in September 2015 for critical care medicine trials in which landmark mortality was the primary outcome. Studies were excluded if they were not two-sided superiority trials, cluster, or cluster crossover trials; if they were focused on a pediatric population; if they were not related to critical care medicine; or if they were purely investigations of surgical techniques. Trials that had completed recruitment were also excluded. Trials registered on more than one database were included only once. Trial investigators were emailed to request data used to inform their sample size calculations. Any trials found to meet exclusion criteria as a result of the reply from their investigators (e.g., trials no longer recruiting) were excluded.

We recorded the following trial characteristics from online registries: sample size, eligibility criteria for trial participants, intervention details, comparison group details (e.g., placebo or usual-care strategy), trial origin country, and landmark for mortality outcome measurement (e.g., 28 days). We recorded the following from investigator replies: power used in sample size calculations, expected baseline mortality, and expected effect size (absolute mortality difference between control and intervention groups).

### Survey

Each trial identified in our systematic review was presented according to the participants, intervention, comparison, outcome standard, and clinicians were asked two questions per trial. First, they were asked to estimate the percentage chance that the actual effect of the treatment being investigated in a particular trial would equal or exceed the effect postulated by investigators. Second, they were asked to specify the largest absolute mortality reduction that they considered to be plausibly attributable to each treatment being investigated. For example, for the ADjunctive coRticosteroid trEatment iN criticAlly ilL Patients with Septic Shock trial [8], which is a 3800-participant trial in which researchers are investigating the effect of a continuous 7-day intravenous infusion of 200 mg/day hydrocortisone on day 90 mortality among adults who are ventilated with septic shock and have received vasopressors/inotropes for at least 4 h, clinicians were asked the following questions:

1. Assuming a baseline day 90 mortality rate of 33% (the baseline mortality rate used by investigators in their power calculations [8]), what do you think the chances are that a continuous 7-day intravenous infusion of 200 mg per day of hydrocortisone reduces absolute mortality by 5% or more? (Answers from 0–100% were allowed.)
2. Assuming a baseline day 90 mortality rate of 33%, what is the largest absolute reduction in day 90 mortality that you believe could occur as a result of a continuous 7-day intravenous infusion of 200 mg per day of hydrocortisone? (Answers from 0% to 33% were allowed.)

Demographic data collected from survey respondents were region of residence (Australia and New Zealand [ANZ], United Kingdom [UK], Europe [outside UK], United States [USA], Canada, Central or South America, Asia, Africa) and training background (intensive care specialist, other specialist, training to be a specialist in intensive care medicine, training in an area of medicine other than intensive care). Intensive care specialists were asked how long they had been working as an intensive care specialist (<5 years, 5–10 years, >10 years).

The survey was piloted by 20 clinicians from ANZ, USA, Europe, and the UK who provided feedback on ease of use, interface, and the survey’s duration. The length of the survey was reduced following the pilot phase because feedback indicated that the original version was too long. Additional file 1 shows the final version of the survey, which was distributed with the weekly *Critical Care Reviews* newsletter over 4 consecutive weeks [9]. The newsletter had 6243 subscribers at the end of this 4-week period. No demographic data are collected from list subscribers; however, the list is free to subscribe to from anywhere in the world, and no restrictions are placed on registration. The email containing the survey was opened by between 2788 and 2889 people per week in each of the 4 weeks in which the survey was running. We chose to “crowd-source” responses from clinicians with an interest in critical care to provide us with “real-world” opinions.

### Outcomes

The primary outcome was the clinicians’ perceptions of prior probability for each trial, which was defined as the percentage chance that each trial would demonstrate a mortality effect equal to or greater than that used in the sample size calculation for that trial.

The following were secondary outcomes:

1. The calculated chance that a statistically significant result at the *P* = 0.05 level for each trial would represent a true-positive
2. The largest effect size that surveyed clinicians considered plausible for each trial
3. The sample size that each trial would require to detect the median largest effect size considered plausible by clinicians

### Statistical analysis

**Fig. 1:**
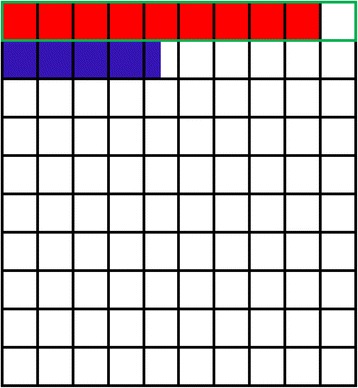
Graphical representation of the method of estimation of the chances that a statistically significant result represents a “true–positive” based on 100 hypothetical trials where there is a 10% chance the hypothesis is correct and experiments are conducted with 90% power at an α of 0.05. In this example, where there is a 10% prior probability that the hypothesis is correct, each box represents a hypothetical trial. The top row of boxes (surrounded by a *green line*) represent the 10 occasions where the hypothesis is correct; the remaining 90 boxes represent the occasions where the null hypothesis is correct. In an experiment with 90% power, one would expect to correctly identify nine of ten correct hypotheses (the area shaded *red*). Because the α value is defined as the probability of rejecting the null hypothesis when the null hypothesis is correct, one would also expect to incorrectly reject the null hypothesis on 4.5 of 90 occasions (the area shaded *blue*). As a result, with a 10% prior probability in an experiment with 90% power, a true-positive result is expected 67% of the time when the *P* value is 0.05

Continuous variables are reported as median and IQR or mean ± SD, and categorical variables are reported as counts and percents. Clinician-perceived prior probability for each trial was used to derive an estimate that a statistically significant result at the *P* = 0.05 level would represent a true-positive using the method described in Fig. 1. Specifically, as outlined in Additional file 2, the chance of a true-positive was calculated as follows [10]:

$$ \mathrm{True}\hbox{-} \mathrm{positive}\ \mathrm{rate} = \left(\mathrm{prior}\ \mathrm{probability} \times \upbeta \right)/\left(\left(1\ \hbox{-}\ \mathrm{prior}\ \mathrm{probability}\right) \times \upalpha \right) + \left(\mathrm{prior}\ \mathrm{probability} \times \upbeta, \Big)\right) $$

The sample size that each trial would require to detect the median largest effect size considered plausible by clinicians was calculated using standard methods for trials designed to compare two binomial proportions. We used the same β for these calculations as investigators had used in their initial sample size calculations and assumed an α of 0.05. Analysis of variance was used to analyze differences in survey results by location and specialty. A Mann-Whitney *U* test was used to compare clinicians’ estimates of effect size, with treatment effect sizes used to inform sample size calculations. A *P* value of <0.05 was considered to indicate statistical significance. Statistical analysis was performed using Real Statistics Resource Pack release 3.8 software (London, UK).

## Results

### Search results

**Fig. 2:**
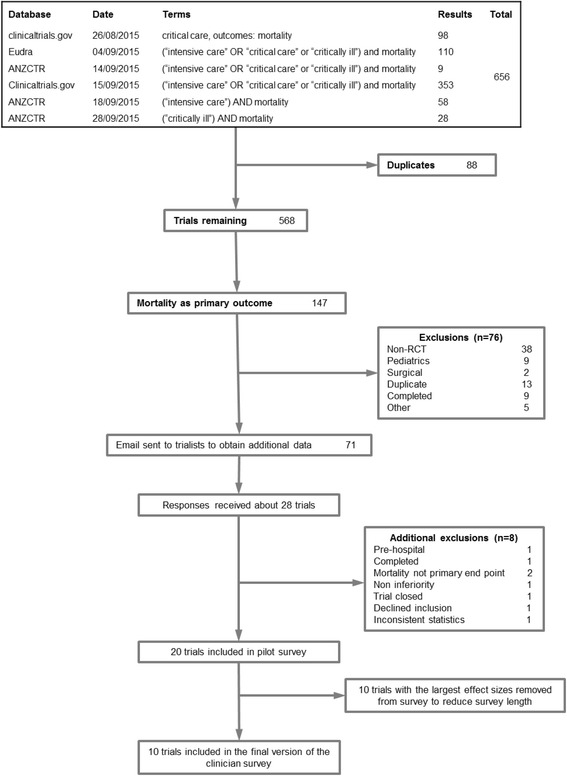
Trials included in the clinician survey. *RCT* Randomized controlled trial

Trial registry searches returned 656 results, and 71 trials met our criteria for the request of further information from investigators. Twenty-eight responses were received, yielding a further eight exclusions and a final set of twenty trials for analysis. All 20 eligible trials were included in the pilot survey, but feedback indicated that the survey was too long; hence, we decided to include only the 10 trials [8, 11–19] with the smallest postulated effect sizes in the final survey (Fig. 2).

### Characteristics of trials included in the survey

Trials included in the clinician survey had a median sample size of 3575 participants (IQR 725–7000), a median baseline mortality rate used in their sample size calculations of 26.5% (IQR 25–33%), and a median postulated treatment effect size of 5% absolute mortality reduction (IQR 3.6–10%) (Table 1).

**Table 1:** Trials included in the clinician survey^a^

Trial name	Participants	Intervention	Comparator	Outcome	Baseline mortality (%)	Postulated mortality effect^b^ (%)	Power (%)	Sample Size
A Confirmatory Phase II/III Study Assessing Efficacy, Immunogenicity and Safety of IC43 Recombinant *Pseudomonas* Vaccine in Intensive Care Patients	Adults requiring ICU ventilation for ≥48 h	Recombinant *Pseudomonas* vaccine	Placebo	Day 28 mortality	27.5	10	90	800
ADjunctive coRticosteroid trEatment iN criticAlly ilL Patients with Septic Shock	Adults who are ventilated with septic shock	Hydrocortisone	Placebo	Day 90 mortality	33	5	90	3800
Early Spontaneous Breathing in ARDS	Adults ventilated with ARDS	APRV	Volume control ventilation	Day 28 mortality	35	10	80	700
Non-sedation versus Sedation with a Daily Wake-up Trial in Critically Ill Patients Receiving Mechanical Ventilation	Adults ventilated in ICU	Nonsedation	Daily awakening	Day 90 mortality	39	10	80	700
Stress Ulcer Prophylaxis in the Intensive Care Unit	Adults with shock, coagulopathy, or receiving RRT or ventilation	Pantoprazole	Placebo	Day 90 mortality	25	5	90	3350
The Augmented versus Routine Approach to Giving Energy Trial	Ventilated adults expected to require enteral nutrition for ≥2 days in ICU	Nutrition at 1.5 kcal/kg/h	Nutrition at 1.0 kcal/kg/h	Day 90 mortality	25	3.95	80	4000
The SuDDICU Cluster RCT of Antibiotic Prophylaxis in Critical Illness	Adults ventilated for ≥24 h in the ICU	SDD	Placebo	In-hospital mortality	25	3.5	90	24,000
Ticagrelor in Severe CAP	Adults with severe CAP requiring ICU admission	Ticagrelor	Placebo	Day 90 mortality	33	11	80	568
Tranexamic acid for the treatment of gastrointestinal haemorrhage: an international randomised, double-blind placebo-controlled trial	Adults with acute significant GI bleeding	Tranexamic acid	Placebo	Day 28 mortality	10	2.5	90	8000
Tranexamic acid for the treatment of significant traumatic brain injury: an international, randomised, double-blind, placebo-controlled trial	Traumatic brain injury	Tranexamic acid	Placebo	Day 28 mortality	20	3	90	10,000

*Abbreviations*: *APRV* Airway pressure release ventilation, *ARDS* Acute respiratory distress syndrome, *CAP* Community-acquired pneumonia, *GI* Gastrointestinal, *ICU* Intensive care unit, *SDD* Selective digestive decontamination, *RRT* Renal replacement therapy

^a^The full description of the participants, intervention, comparator, and outcomes provided in the survey is shown in Appendix 1 in Additional file 2; brief information provides an overview of the included trials

^b^Postulated mortality effect is the investigator-specified absolute risk reduction in mortality used in sample size calculations

### Survey results

#### Responses

Completed responses were received from 166 (2.7%) of 6243 *Critical Care Reviews* subscribers.

#### Demographics

Of all respondents, 37 (22.3%) were based in the USA, 47 (28.3%) in the UK, and 29 (17.5%) in ANZ. The majority (101 [60.8%]) were ICU specialists, 46 (45.5%) of whom had less than 5 years of experience at this level and 26 (25.7%) of whom had more than 10 years of experience at this level (*see* Additional file 2: Table S1).

#### Probabilities, effect size, and sample size

Clinicians’ estimates of prior probability varied very widely, from 0% to 100% for most trials, with a median trial prior probability of 15% (IQR 10–20%) (Table 2 and Additional file 2: Figure S1). On the basis of these estimates, the median estimate of probability of a true-positive result for each trial was 73.5% (IQR 64–82%) (probability of true-positive results derived as per Fig. 1); however, for every trial, the estimated chance of a true-positive was between 0% and 99% or 100% when the full range of estimates of perceived prior probability provided by survey respondents was considered (Table 2 and Additional file 2: Figure S1).

**Table 2:** Prior probability estimates and calculated chances of a true positive result for each trial

Trial name	Prior probability estimates^a^ (%)		Chance of a true-positive^b^ (%)	
	Median estimate (IQR)	Range of estimates	Median calculated chance (IQR)	Range of calculated chances
A Confirmatory Phase II/III Study Assessing Efficacy, Immunogenicity and Safety of IC43 Recombinant *Pseudomonas* Vaccine in Intensive Care Patients	5 (0.33–20)	0–82	49 (6–82)	0–99
ADjunctive coRticosteroid trEatment iN criticAlly ilL Patients with Septic Shock	20 (5–50)	0–100	82 (49–95)	0–100
Early Spontaneous Breathing in ARDS	10 (1–25)	0–100	64 (14–84)	0–100
Non-sedation versus Sedation with a Daily Wake-up Trial in Critically Ill Patients Receiving Mechanical Ventilation	20 (5–40)	0–100	80 (46–91)	0–100
Stress Ulcer Prophylaxis in the Intensive Care Unit	10 (0.030–28.75)	0–100	67 (1–88)	0–100
The Augmented versus Routine Approach to Giving Energy Trial	10 (2–40)	0–90	64 (29–91)	0–99
The SuDDICU Cluster RCT of Antibiotic Prophylaxis in Critical Illness	25 (10–50)	0–100	86 (67–95)	0–100
Ticagrelor in Severe CAP	2.5 (0–10)	0–100	29 (0–64)	0–100
Tranexamic acid for the treatment of gastrointestinal haemorrhage: an international randomised, double-blind, placebo-controlled trial	35 (10–57.5)	0–100	91 (67–96)	0–100
Tranexamic acid for the treatment of significant traumatic brain injury: an international, randomised, double-blind, placebo-controlled trial	20 (10–50)	0–100	82 (67–95)	0–100

*Abbreviations*: *APRV* Airway pressure release ventilation, *ARDS* Acute respiratory distress syndrome, *CAP* Community-acquired pneumonia, *GI* Gastrointestinal, *ICU* Intensive care unit, *IQR* Interquartile range, *SDD* Selective digestive decontamination, *RRT* Renal replacement therapy

^a^Prior probability was defined as the percentage chance, estimated by clinicians, that a trial would demonstrate a mortality effect equal to or greater than that used by the trials investigators in their sample size calculation

^b^Calculated chances of a true-positive result are based on the prior probabilities shown and assume power (β) specified by the trial investigators and an α of 0.05 using the following calculation: True-positive rate = (prior probability) × β/(1 − prior probability) × α) + (prior probability × β)

The median largest absolute mortality reduction considered plausible was 4.5% (IQR, 3.5% to 5%) compared with a median absolute mortality reduction used in sample size calculations of 5% (IQR, 3.6% to 10%) (*P* = 0.27) (Table 3 and Additional file 2: Figure S2, Online Data Supplement). For three trials [8, 11, 12], the actual trial sample size was greater than that needed to detect the median largest effect size considered plausible by survey respondents. For six trials [14–17, 19] sample sizes were too small to detect the median largest effect size considered plausible, often by more than 2000 participants.

**Table 3:** Largest effect size considered plausible by clinicians and the corresponding sample size to detect this effect

Trial name	Effect size (%)		Sample size	
	Median largest absolute mortality reduction considered plausible (IQR, range)	Absolute mortality reduction postulated by trialists	Size required to detect median largest effect size considered plausible	Actual
A Confirmatory Phase II/III Study Assessing Efficacy, Immunogenicity and Safety of IC43 Recombinant *Pseudomonas* Vaccine in Intensive Care Patients	5 (2–6, 0–23))	10	3186	800
ADjunctive coRticosteroid trEatment iN criticAlly ilL Patients with Septic Shock	5 (3–10, 0–33)	5	3556	3800
Early Spontaneous Breathing in ARDS	5 (2–10, 0–35)	10	2748	700
Non-sedation versus Sedation with a Daily Wake-up Trial in Critically Ill Patients Receiving Mechanical Ventilation	5 (3–15, 0–39)	10	2904	700
Stress Ulcer Prophylaxis in the Intensive Care Unit	3 (1–5, 0–25)	5	8388	3350
The Augmented versus Routine Approach to Giving Energy Trial	3 (1–5, 0–25)	3.95	6266	4000
The SuDDICU Cluster RCT of Antibiotic Prophylaxis in Critical Illness	5 (3–9, 0–24)	3.5	Not calculated^a^	24,000
Ticagrelor in Severe CAP	3 (1–5, 0–33)	11	7522	568
Tranexamic acid for the treatment of gastrointestinal haemorrhage: an international randomised, double-blind, placebo-controlled trial	3 (2–5, 0–10)	2.5	3624	8000
Tranexamic acid for the treatment of significant traumatic brain injury: an international, randomised, double-blind, placebo-controlled trial	4 (2–5, 0–10)	3	3868	10,000

*Abbreviations*: *APRV* Airway pressure release ventilation, *ARDS* Acute respiratory distress syndrome, *ARR* Absolute risk reduction, *CAP* Community-acquired pneumonia, *GI* Gastrointestinal, *ICU* Intensive care unit, *IQR* Interquartile range, *SDD* Selective digestive decontamination, *RRT* Renal replacement therapy

^a^Data required to perform the modified sample size calculation for this cluster trial could not be derived from the survey response data

## Discussion

### Statement of principal findings

We conducted a systematic review of trial registries to identify ongoing trials in the field of critical care medicine in which researchers are reporting landmark mortality as the primary outcome. We then conducted a clinician survey to establish views about the prior probability that the interventions in ten of these trials would reduce mortality by at least as much as postulated by investigators. Moreover, we also sought to establish clinicians’ estimates of the largest plausible mortality reduction that might be attributable to each study intervention. We found that, in aggregate, respondents’ estimates of prior probability were low, but we also found that individual estimates varied widely, from 0% to 100%, for most trials. We also found that the median largest absolute reduction in mortality considered plausible was ≤5% for all study interventions. Although some trials were powered to detect such effect sizes, many were underpowered to detect effects of this magnitude by more than 2000 participants.

### Study significance

This study represents the first attempt to provide quantitative estimates of clinicians’ perceptions about prior probability and plausible effect sizes for ongoing trials in the field of critical care medicine. Researchers in a number of previous studies have systematically evaluated rates of reported positive results in trials of critically ill patients with mortality endpoints. In one study, researchers reported positive results in 10 (14%) of 72 multicenter RCTs with mortality as the primary endpoint published before August 2006 [20]; in a second, investigators reported that 7 (18%) of 38 trials published in 5 major medical journals between 1999 and 2009 showed positive results [5]; and in a third study, in evaluating ICU-based trials published between January 2007 and May 2013 in 16 high-impact general or critical care journals, researchers identified that 3 (9%) of 34 were positive [21]. Authors of a more recent systematic review identified that 44 (5%) of 862 multicenter critical care medicine trials reported significant differences in mortality [22]. These data confirm that ICU-based trials with mortality endpoints are frequently negative and indicate that the median predictions of prior probability offered by survey respondents in our study are broadly congruent with the observed frequency of positive trials in the critical care medicine literature. However, they do not necessarily support the accuracy of the estimates of low prior probability provided for the ten large trials included in our survey. Logically, the accuracy of such estimates can only be determined prospectively by comparing prior probabilities and actual trial results for a large number of trials over time.

Our method of eliciting priors through clinician survey is importantly different from other ways of eliciting priors in that it is a pragmatic, “real-world” method employing actual end-users of the trials to be assessed, whose beliefs ultimately will decide the impact of the trials on their practice. Previous work has used abstract modeling or “experts” (i.e., generators of research rather than end-users) [23, 24].

The extreme variability of the estimates, coupled with some manifestly implausible responses (e.g., suggestions that particular treatments might reduce mortality by 100%), could be interpreted as an indication that our estimates lack validity. However, outlier responses have a limited effect on estimates based on medians, and the clinical equipoise required for the initiation of a trial [25] might reasonably be expected to result in a range of estimates from the members of clinical community. That said, our finding that effect sizes postulated by investigators often appear to be larger than the median effect sizes considered plausible by clinicians is consistent with previous literature suggesting that effect sizes used to inform sample size calculations are often inflated [5, 21]. For the range of interventions being tested in the studies in our survey, the largest treatment-associated mortality differences considered plausible would not be excluded by the 95% CIs in the vast majority of the 40 trials with a primary endpoint of mortality identified in a recent systematic review of high-impact critical care-based trials [21]. Only six superiority trials identified in this systematic review had ≥80% power to detect a treatment-associated mortality reduction of ≤5% [21].

### Strengths and weaknesses

The key strength of our study lies in the fact we used data from sample size calculations for ongoing critical care trials to evaluate clinician estimates of prior probability and plausibility in a way that had not been attempted previously. The systematic approach of searching trial registries ensured that relevant and important trials were captured. Because our sample of trials was small, there is a high risk of both type I and type II errors in our results, and consequently our analysis should be considered hypothesis-generating. Moreover, because we included only the trials with the smallest postulated effect sizes in our survey, our results are unlikely to be representative of what would be found if all currently recruiting trials were considered. Our sample was chosen in this way to allow assessment of those trials with the most plausible (or achievable) *prima facie* effect sizes, giving us results for a model set of the “best” critical care trials. Because we depended on trialists responding to our queries regarding their sample size calculations, there was additional selection bias applied to the trials we evaluated. Respondents were not blinded to trialists’ postulated effect sizes, and knowledge of these may have biased their responses.

Although our survey response rate was low (2.7%), a sufficient number of responses was achieved to provide broad geographical representation among respondents. In the descriptions of trials used in our survey, we asked respondents to assume that estimates of control arm mortality used by investigators were accurate. However, it appears that control arm mortality rates are often overestimated in critical care medicine trials [21]. We chose not to alter control arm mortality rates in our survey, because doing so would have added substantial complexity to the scenarios being considered. On the one hand, as the control arm mortality rate falls, the proportion of potentially salvageable patients would be expected to fall, making the same absolute mortality reductions less likely. However, on the other hand, as the control arm mortality rate moves away from 50%, the power to detect given absolute differences increases.

Our approach to determining the chances of a true-positive for each trial provides only a point estimate and does not account for the true distribution of probability estimates [26]. For our calculations, we assumed a *P* value of 0.05. If lower *P* values were observed, this would lead to correspondingly higher probabilities of a true-positive result. Our approach was chosen because it provides estimates that are likely to be readily understood by clinicians. The probability of a true-positive result for a given trial that should be accepted or rejected is not established, but logically this should depend on the particular treatment being considered. For comparisons between standard treatments with similar known risk profiles and similar costs, the threshold value for practice change should probably be lower than for expensive new treatments, where risk profiles are less certain.

### Implications for clinical practice

The low perceived prior probabilities and exaggerated effect sizes suggested by our results are potentially of concern to clinicians who will need to interpret the results of these trials when they are completed. Rejecting the null hypothesis in favor of the experimental hypothesis on the basis of a *P* value threshold of 0.05 in the setting of low prior probability will potentially result in clinicians and investigators drawing erroneous conclusions [26, 27]. If clinicians’ perceptions of low prior probabilities are correct, then the predominance of low prior probability in hypotheses being evaluated may explain the frequent failure to replicate positive results in critical care medicine trials [28] and in trials in other disciplines [29]. However, as we have highlighted, the accuracy of the estimates of prior probability and effect size provided by our survey respondents is unknown. Nevertheless, our study is an important first step toward developing more robust assessments of prior probability in the future.

## Conclusions

Our study represents the first attempt to provide quantitative estimates of clinicians’ opinions about prior probability and plausibility of effect sizes for trials in the field of critical care medicine. Our preliminary data indicate that, even for some of the largest trials currently recruiting, many clinicians appear to regard prior probabilities as low and consider that the plausible effects on absolute mortality for study treatments being investigated are ≤5%. This finding suggests that future trials with a primary endpoint of landmark mortality should, in general, be powered to detect absolute mortality differences <5% and those that are not are, until proven otherwise, likely to be considered underpowered by clinicians.

Estimates of prior probability are vitally important to the proper interpretation of a trial’s results. Consequently, we recommend that trialists consider providing estimates of prior probability in their prepublished statistical analysis plans [30]. Further work is needed to determine whether pooled estimates obtained by “crowd-sourcing” clinicians’ views of perceived prior probability via survey provide a replicable and accurate method of assessing prior probability or whether other methods of estimating prior probability [31] are preferred.

## Additional files

Additional file 1: Survey.pdf (final version of survey). (PDF 113 kb)

Additional file 2: Online data supplement. (DOCX 473 kb)
