# Supplementary material for: Effect sizes in ongoing randomized controlled critical care trials
Source: Crit Care. 2017 Jun 5;21:132. doi: 10.1186/s13054-017-1726-x (PMC5460326; doi:10.1186/s13054-017-1726-x)
Supplement: Supplementary file 1 — Survey.pdf (final version of survey). (PDF 113 kb) [file 13054_2017_1726_MOESM1_ESM.pdf]

Introduction

Many thanks for taking part in this survey evaluating effect sizes in currently active trials in critically ill patients.

You will be shown information on 10 trials, and you will be asked two questions about each.

If you are unfamiliar with an intervention in a trial, please make a best-guess at the effect size based on the information given.

Your answers are anonymous, and by completing this survey, you agree that the information you provide can be used as part of a dataset for academic publication and presentation.

Dr Elliott Ridgeon
Dr Rachel Varughese
Dr Scott Aberegg
Prof Rinaldo Bellomo
Dr Landoni Giovanni
Dr Rob MacSweeney
Dr Paul Young

On behalf of the Medical Research Institute of New Zealand

Demographics

1. Which country or region do you work in?

- ANZ
- Europe (outside UK)
- UK
- USA
- Canada
- Central or South America
- Asia
- Africa

2. What is your stage of training in Intensive Care Medicine?

- Intensive Care Specialist
- Other Specialist
- Training to be a Specialist in Intensive Care Medicine
- Training in an area of medicine other than Intensive Care Medicine

3. How many years experience do you have as a Specialist in Intensive Care Medicine?

- Less than 5 years
- 5 to 10 years
- More than 10 years

Trial 1

A CONFIRMATORY PHASE II/III STUDY ASSESSING EFFICACY, IMMUNOGENICITY AND SAFETY OF IC43 RECOMBINANT PSEUDOMONAS VACCINE IN INTENSIVE CARE PATIENTS.

Population: Adults requiring ICU ventilation for at least 48h

Intervention: Recombinant pseudomonas vaccine, given IM at day 0 (enrolment) and again at day 7.

Comparator: Placebo

Outcomes: Mortality at 28 days

4. Assuming a baseline day 28 mortality rate of 27.5%, what do you think the chances are that recombinant pseudomonas vaccine reduces absolute mortality by 10% or more (i.e. a decrease in mortality from 27.5% to 17.5%)?

Enter a value between 0 and 100%

5. Assuming a baseline day 28 mortality rate of 28%, what is the largest absolute reduction in day 28 mortality that you believe could occur as the result of administration of a recombinant pseudomonas vaccine?

- I don't know
- **Enter a value between 0 and 28%**

Trial 2

ADJUNCTIVE CORTICOSTEROID TREATMENT IN CRITICALLY ILL PATIENTS WITH SEPTIC SHOCK

Population: Adults who are ventilated with septic shock and have received vasopressors/inotropes for at least 4 hours

Intervention: Intravenous hydrocortisone (continuous infusion of 200mg/day for 7 days)

Comparator: Placebo

Outcomes: Mortality at 90 days

6. Assuming a baseline day 90 mortality rate of 33%, what do you think the chances are that intravenous hydrocortisone infusion reduces absolute mortality by 5% or more?

Enter a value between 0 and 100%

7. Assuming a baseline day 90 mortality rate of 33%, what is the largest absolute reduction in day 90 mortality that you believe could occur as the result of administration of intravenous hydrocortisone (continuous infusion of 200mg/day for 7 days)?

- I don't know
- **Enter a value between 0 and 33%**

Trial 3

EARLY SPONTANEOUS BREATHING IN ACUTE RESPIRATORY DISTRESS SYNDROME

Population: Ventilated adults with ARDS

Intervention: Airway Pressure Release Ventilation (APRV) to allow spontaneous breathing cycles within the acute phase of ARDS

Comparator: Volume controlled ventilation

Outcomes: 28 day mortality

8. Assuming a baseline in-hospital mortality rate of 35%, what do you think the chances are that APRV in the acute phase of ARDS reduces absolute mortality by 10% or more?

Enter a value between 0 and 100%

[ ]

9. Assuming a baseline in-hospital mortality rate of 35%, what is the largest absolute reduction in in-hospital mortality that you believe could occur as the result of using Airway Pressure Release Ventilation (APRV) to allow spontaneous breathing cycles within the acute phase of the disease?

- ◯ I don't know
- ◯ **Enter a value between 0 and 35%**

[ ]

Trial 4

NON-SEDATION VERSUS SEDATION WITH A DAILY WAKE-UP TRIAL IN CRITICALLY ILL PATIENTS RECEIVING MECHANICAL VENTILATION

Population: Adults who are ventilated on the intensive care unit (without severe head trauma or status epilepticus)

Intervention: Non-sedation. Patients are informed by staff of where they are, what has happened, and what type of treatment they are going to receive. Participants will be awake and have a natural sleep rhythm. Patients with delirium will be calmed by staff or treated with haloperidol

Comparator: Sedation with daily wake-up. Sedation to Ramsay score 3-4. First 48 hours sedation with propofol, after 48 hours midazolam will be used. During daytime, the patient will be awakened.

Outcomes: 90 day mortality

10. Assuming a baseline day 90 mortality rate of 39%, what do you think the chances are that a non-sedation policy reduces absolute mortality by 10% or more?

Enter a value between 0 and 100%

[ ]

11. Assuming a baseline day 90 mortality rate of 39%, what is the largest absolute reduction in day 90 mortality that you believe could occur as the result of adopting a policy of non-sedation?

- ○ I don't know
- ○ **Enter a value between 0 and 39%**

  [ ]

Trial 5

STRESS ULCER PROPHYLAXIS IN THE INTENSIVE CARE UNIT

Population: Adults with shock, coagulopathy, or receiving continuous renal replacement therapy or ventilation

Intervention: IV pantoprazole (40mg/day for duration of ICU stay)

Comparator: Placebo

Outcomes: 90 day mortality

12. Assuming a baseline day 90 mortality rate of 25%, what do you think the chances are that IV pantoprazole reduces absolute mortality by 5% or more?

Enter a value between 0 and 100%

13. Assuming a baseline 90 day mortality rate of 25%, what is the largest absolute reduction in 90 day mortality that you believe could occur as the result of administration of IV pantoprazole (40mg/day for duration of ICU stay)?

- I don't know
- **Enter a value between 0 and 25%**

Trial 6

THE AUGMENTED VERSUS ROUTINE APPROACH TO GIVING ENERGY TRIAL (TARGET)

Population: Ventilated adults expected to require enteral (not oral) nutrition for at least 2 days in ICU

Intervention: Nutrition at 1.5kcal/kg/hr (given for 28 days or until ICU discharge, whichever is sooner)

Comparator: Nutrition at 1.0kcal/kg/hr (given for 28 days or until ICU discharge, whichever is sooner)

Outcomes: 90 day mortality

14. Assuming a baseline 90 day mortality rate of 25%, what do you think the chances are that nutrition at 1.5kcal/kg/hr (instead of at 1kcal/kg/hr) reduces absolute mortality by 3.95% or more?

Enter a value between 0 and 100%

15. Assuming a baseline 90 day mortality rate of 25%, what is the largest absolute reduction in 90 day mortality that you believe could occur as the result of administration of nutrition at 1.5kcal/kg/hr instead of at 1kcal/kg/hr?

- ○ I don't know
- ○ **Enter a value between 0 and 25%**

Trial 7

THE SUDDICU STUDY OF ANTIBIOTIC PROPHYLAXIS IN CRITICAL ILLNESS

Population: Adults ventilated for more than 24 hours in the ICU

Intervention: Triple selective decontamination of the digestive tract (antibiotic paste to buccal mucosa/oropharynx, antibiotic suspension to GI tract and IV antibiotic)

Comparator: Usual care (as per local VAP prevention guidelines)

Outcomes: In-hospital mortality

16. Assuming a baseline in-hospital mortality rate of 25%, what do you think the chances are that using triple selective decontamination of the digestive tract reduces absolute mortality by 3.5% or more?

Enter a value between 0 and 100%

17. Assuming a baseline in-hospital mortality rate of 25%, what is the largest absolute reduction in in-hospital mortality that you believe could occur as the result of using triple selective decontamination of the digestive tract (antibiotic paste to buccal mucosa/oropharynx, antibiotic suspension to GI tract and IV antibiotic)?

- I don't know
- **Enter a value between 0 and 25%**

Trial 8

TICAGRELOR IN SEVERE COMMUNITY ACQUIRED PNEUMONIA

Population: Adults with new severe community acquired pneumonia requiring ICU admission

Intervention: Ticagrelor (180mg loading dose then 90mg twice daily for 90 days)

Comparator: Placebo

Outcomes: 90 day mortality

18. Assuming a baseline 90 day mortality rate of 33%, what do you think the chances are that ticagrelor reduces absolute mortality by 11% or more?

Enter a value between 0 and 100%

19. Assuming a baseline 90 day mortality rate of 33%, what is the largest absolute reduction in 90 day mortality that you believe could occur as the result of administration of ticagrelor (180mg loading dose then 90mg twice daily for 90 days)?

- I don't know
- **Enter a value between 0 and 33%**

Trial 9

TRANEXAMIC ACID FOR THE TREATMENT OF GASTROINTESTINAL HAEMORRHAGE: AN INTERNATIONAL RANDOMISED, DOUBLE BLIND PLACEBO CONTROLLED TRIAL

Population: Adults with acute significant GI bleeding (upper or lower)

Intervention: Tranexamic acid (loading dose of 1g IV, then 3g IV infusion over 24h)

Comparator: Placebo

Outcomes: 28 day mortality

20. Assuming a baseline 28 day mortality rate of 10%, what do you think the chances are that tranexamic acid reduces absolute mortality by 2.5% or more?

Enter a value between 0 and 100%

[ ]

21. Assuming a baseline 28 day mortality rate of 10%, what is the largest absolute reduction in 28 day mortality that you believe could occur as the result of administration of tranexamic acid (loading dose of 1g IV, then 3g IV infusion over 24h)?

- ○ I don't know
- ○ **Enter a value between 0 and 10%**

[ ]

Trial 10

TRANEXAMIC ACID FOR THE TREATMENT OF SIGNIFICANT TRAUMATIC BRAIN INJURY: AN INTERNATIONAL, RANDOMISED, DOUBLE BLIND, PLACEBO CONTROLLED TRIAL.

Population: Adults with acute traumatic brain injury (with intracranial bleeding or GCS<12)

Intervention: Tranexamic acid (loading dose of 1g IV, then 1g IV infusion over 8h)

Comparator: Placebo

Outcomes: 28 day mortality

22. Assuming a baseline 28 day mortality rate of 20%, what do you think the chances are that tranexamic acid reduces absolute mortality by 3% or more?

Enter a value between 0 and 100%

[ ]

23. Assuming a baseline 28 day mortality rate of 20%, what is the largest absolute reduction in 28 day mortality that you believe could occur as the result of administration of tranexamic acid (loading dose of 1g IV, then 1g IV infusion over 8h)?

- ○ I don't know
- ○ **Enter a value between 0 and 20%**

[ ]

Trials Complete!

Many thanks for participating in this survey about effect sizes in critical care trials.

If you have any feedback about this project, please leave your comments below.

24. Feedback and Comments
