# Supplementary material for: Effect sizes in ongoing randomized controlled critical care trials
Source: Crit Care. 2017 Jun 5;21:132. doi: 10.1186/s13054-017-1726-x (PMC5460326; doi:10.1186/s13054-017-1726-x)
Supplement: Supplementary file 2 — Online data supplement. (DOCX 473 kb) [file 13054_2017_1726_MOESM2_ESM.docx]

**Online data supplement**

**Supplemental methods & results:**

*Calculation of the true positive rate*

In this study we sought to determine the true positive rate (TPR) of hypothetical trials based on estimates of prior probability (Pr). Frequentist statistical theory does not specifically address prior Pr. Instead, the null hypothesis (HO) and the alternative hypothesis (HA) are considered unknown. In order to determine the TPR we used methods previously described by Wacholder et al [10]. In brief, we considered HO and HA probabilistically. A statistical test T has statistical size α for testing H0 when rejection of H0 is defined as T>zα and Pr(T>zα | H0 is true) = Pr(rejecting H0 | HA is false) = α. Statistical power is denoted by 1 − β, with Pr(T>zα | H0 is false) = Pr(rejecting H0 | HA is true) = 1 − β or the probability of rejecting when the alternative hypothesis HA is true. We define false positive rate for standard statistical significance testing as Pr (H0 is true | association is deemed statistically significant) = Pr(H0 is true | T>zα), where zα is the α of the standard normal distribution. Thus:

TPR=((prior Pr) x β) /( (1-prior Pr) x α level) + (prior Pr x β))

*Survey Results – Differences by geography and training*

Demographics of survey respondents are described in Table S1.

The mean estimates of perceived prior probability that a recombinant *Pseudomonas* vaccine would reduce absolute mortality in adults requiring ≥48 hours of mechanical ventilation by ≥10% varied significantly by geographic location (P<0.01) with the highest prior probability estimate of 44% in respondents from Africa and the lowest estimate of 3.5% in respondents from Canada. There were no other significant differences in estimates of prior probability by location observed (Table S2).

The mean largest effect size considered plausible varied by geographic location for trials investigating steroids in septic shock, selective digestive decontamination, and tranexamic acid in traumatic brain injury but not for other trials included in the survey (Table S3).

The perceived prior probability of demonstrating an absolute mortality difference of greater than or equal to that postulated by trial investigators varied by training for trials of early spontaneous breathing in acute respiratory distress syndrome, energy-dense enteral nutrition, and tranexamic acid in gastrointestinal haemorrhage with intensive care specialists providing lower estimates of prior probability than other groups for all three of these trials (Table S4). However, the largest effect size considered plausible for each trial did not differ significantly by training (Table S5).

*Supplemental Tables and Figures*

| **Table S1. Characteristics of survey respondents (n=166)** | |
| --- | --- |
| **Characteristic** | **Number (%)** |
| Geographic region of residence |  |
| ANZ | 29 (17.5) |
| UK | 48 (28.3) |
| Europe | 24 (14.5) |
| USA | 37 (22.3) |
| Africa | 3 (1.8) |
| Canada | 6 (3.6) |
| Asia | 15 (9.0) |
| Central or South America | 5 (3.0) |
| Specialty |  |
| ICU Specialist | 101 (60.8) |
| <5y experience | 46 (45.4) |
| 5-10y experience | 29 (28.7) |
| >10y experience | 26 (25.7) |
| Other Specialist | 21 (12.7) |
| Training to be a Specialist in Intensive Care Medicine | 29 (17.5) |
| Training in an area of medicine other than Intensive Care | 15 (9.0) |

*Abbreviations: ANZ: Australia and New Zealand; ICU: Intensive Care Unit; UK: United Kingdom; USA: United States of America*

| **Table S2. Prior probability^*^ estimates by location** | | | | | | | | | | |
| --- | --- | --- | --- | --- | --- | --- | --- | --- | --- | --- |
| **Trial name** | **Trial origin** | **Mean+/-SD prior probability estimates by location (%)** | | | | | | | | **P-value†** |
|  |  | **ANZ (n=29)** | **Africa (n=3)** | **Asia (n=15)** | **Canada (n=6)** | **Central or South America (n=5)** | **Europe (outside UK) (n=24)** | **UK (n=48)** | **USA (n=37)** |  |
| A Confirmatory Phase II/III Study Assessing Efficacy, Immunogenicity And Safety Of Ic43 Recombinant Pseudomonas Vaccine In Intensive Care Patients | Europe | 7.8  ±12.1 | 44.0  ±41.3 | 21.7  ±20.6 | 3.5  ±3.9 | 17.4  ±11.0 | 13.0  ±19.4 | 8.85  ±13.6 | 13.3  ±15.2 | <0.01 |
| ADjunctive coRticosteroid trEatment iN criticAlly ilL Patients With Septic Shock | ANZ | 17.1  ±15.3 | 40.0  ±45.8 | 26.9± 20.5 | 8.7  ±7.8 | 32.8  ±40.7 | 28.5  ±29.5 | 30.6  ±22.0 | 32.3  ±27.8 | 0.10 |
| Early Spontaneous Breathing in ARDS | Europe | 11.7  ±18.7 | 17.3  ±28.3 | 21.4  ±20.5 | 3.0  ±3.9 | 19.6  ±20.3 | 23.3  ±28.3 | 22.7  ±27.8 | 16.7  ±18.3 | 0.32 |
| Non-sedation Versus Sedation With a Daily Wake-up Trial in Critically Ill Patients Receiving Mechanical Ventilation | Europe | 17.1  ±20.3 | 26.7  ±30.6 | 35.8  ±26.7 | 33.0  ±36.8 | 20.6  ±20.5 | 26.7  ±28.1 | 26.7  ±28.2 | 26.8  ±25.0 | 0.53 |
| Stress Ulcer Prophylaxis in the Intensive Care Unit | Europe | 11.7  ±15.8 | 30.0  ±26.5 | 27.6  ±28.6 | 9.7  ±19.8 | 24.6  ±16.1 | 20.4  ±29.6 | 16.4  ±21.5 | 18.5  ±23.8 | 0.41 |
| The Augmented Versus Routine Approach to Giving Energy Trial | ANZ | 17.2  ±20.5 | 31.7  ±23.6 | 36.8  ±26.8 | 3.2  ±4.0 | 16.0  ±14.7 | 18.9  ±21.4 | 20.6  ±23.1 | 22.2  ±22.4 | 0.06 |
| The SuDDICU Cluster RCT of Antibiotic Prophylaxis in Critical Illness | ANZ | 34.0  ±26.7 | 73.3  ±15.3 | 29.4  ±25.7 | 23.3  ±38.2 | 45.0  ±28.3 | 30.3  ±32.3 | 39.9  ±29.9 | 31.9  ±26.1 | 0.20 |
| Ticagrelor in Severe CAP | UK | 6.2  ±14.7 | 25.0  ±43.3 | 13.8  ±17.1 | 0.7  ±0.8 | 5.0  ±8.7 | 7.8  ±12.3 | 11.6  ±20.3 | 15.3  ±25.2 | 0.32 |
| Tranexamic acid for the treatment of gastrointestinal haemorrhage: an international randomised, double blind placebo controlled trial | UK | 27.2  ±22.2 | 61.7  ±46.5 | 38.2  ±31.1 | 22.5  ±24.7 | 30.0  ±10.0 | 42.8  ±31.9 | 40.1  ±26.3 | 44.0  ±28.1 | 0.12 |
| Tranexamic Acid for the treatment of significant traumatic brain injury: an international, randomised, double blind, placebo controlled trial. | UK | 20.1  ±18.9 | 16.7  ±20.8 | 27.4  ±27.5 | 27.8  ±40.0 | 57.0  ±9.7 | 30.5  ±30.6 | 31.8  ±22.2 | 26.9  ±26.0 | 0.12 |

Abbreviations: ANZ: Australia and New Zealand; APRV: airway pressure release ventilation; ARDS: acute respiratory distress syndrome; CAP: Community Acquired Pneumonia; GI: gastrointestinal; ICU: Intensive Care Unit; SDD: selective digestive decontamination; RRT: renal replacement therapy; UK: United Kingdom

* Prior probability was defined as the percentage chance that a trial would demonstrate a mortality effect equal to or larger than that used by the trials investigators in their sample size calculation.

† P-value calculated by ANOVA

| **Table S3. Largest effect size considered plausible by location** | | | | | | | | | | |
| --- | --- | --- | --- | --- | --- | --- | --- | --- | --- | --- |
| **Trial name** | **Trial origin** | **Mean+/-SD largest effect size considered plausible by location (%)** | | | | | | | | **P-value*** |
|  |  | **ANZ (n=29)** | **Africa (n=3)** | **Asia (n=15)** | **Canada (n=6)** | **Central or South America (n=5)** | **Europe (outside UK) (n=24)** | **UK (n=48)** | **USA (n=37)** |  |
| A Confirmatory Phase II/III Study Assessing Efficacy, Immunogenicity And Safety Of Ic43 Recombinant Pseudomonas Vaccine In Intensive Care Patients | Europe | 6.7  ±8.0 | 17.0 ±4.2 | 12.2 ±9.0 | 3.4  ±2.7 | 9.0  ±2.6 | 4.7  ±6.1 | 6.1  ±9.1 | 6.7  ±6.8 | 0.11 |
| ADjunctive coRticosteroid trEatment iN criticAlly ilL Patients With Septic Shock | ANZ | 8.7  ±8.8 | 6.5  ±2.1 | 10.8  ±9.3 | 4.0  ±3.5 | 31.3 ±46.6 | 10.9 ±14.2 | 6.8  ±4.9 | 9.8  ±9.2 | <0.01 |
| Early Spontaneous Breathing in ARDS | Europe | 6.4  ±6.6 | 2.0  ±0.0 | 9.6 ±11.2 | 3.1  ±1.9 | 14.4 ±20.8 | 12.4 ±22.4 | 6.9  ±6.7 | 8.8  ±9.5 | 0.46 |
| Non-sedation Versus Sedation With a Daily Wake-up Trial in Critically Ill Patients Receiving Mechanical Ventilation | Europe | 8.4  ±9.4 | 27.0 ±17.0 | 15.4 ±10.8 | 7.0  ±5.8 | 16.8 ±22.4 | 11.1 ±12.8 | 9.1  ±8.8 | 10.4 ±10.9 | 0.14 |
| Stress Ulcer Prophylaxis in the Intensive Care Unit | Europe | 5.6  ±6.8 | 5.0  ±0.0 | 6.2  ±6.2 | 4.3  ±6.0 | 13.4 ±10.7 | 4.8  ±6.0 | 3.8  ±4.0 | 7.0  ±10.6 | 0.17 |
| The Augmented Versus Routine Approach to Giving Energy Trial | ANZ | 4.6  ±6.0 | 6.0  ±5.7 | 7.2  ±7.3 | 1.7  ±2.1 | 7.5  ±8.7 | 5.1  ±7.1 | 4.5  ±4.9 | 9.5  ±13.5 | 0.20 |
| The SuDDICU Cluster RCT of Antibiotic Prophylaxis in Critical Illness | ANZ | 6.6  ±5.8 | 20.0  ±0.0 | 8.5  ±9.0 | 3.1  ±2.6 | 11.6  ±6.3 | 5.3  ±5.2 | 5.9  ±4.1 | 6.0  ±5.5 | 0.04 |
| Ticagrelor in Severe CAP | UK | 6.3  ±9.6 | 25.0 ±0.0 | 6.4 ±10.0 | 3.3  ±1.5 | 1.3  ±2.5 | 4.2  ±7.6 | 6.0  ±7.1 | 8.3  ±10.1 | 0.24 |
| Tranexamic acid for the treatment of gastrointestinal haemorrhage: an international randomised, double blind placebo controlled trial | UK | 3.1  ±2.1 | 5.0  ±0.0 | 4.3  ±2.9 | 2.5  ±1.7 | 8.6  ±4.1 | 7.7 ±17.4 | 3.5  ±2.4 | 6.0  ±5.8 | 0.31 |
| Tranexamic Acid for the treatment of significant traumatic brain injury: an international, randomised, double blind, placebo controlled trial. | UK | 5.2  ±4.8 | 5.0  ±0.0 | 4.5  ±4.5 | 3.0  ±2.3 | 14.4  ±15.1 | 5.6  ±5.6 | 4.3  ±3.4 | 6.5  ±7.3 | 0.03 |

Abbreviations: ANZ: Australia and New Zealand; APRV: airway pressure release ventilation; ARDS: acute respiratory distress syndrome; CAP: Community Acquired Pneumonia; GI: gastrointestinal; ICU: Intensive Care Unit; SDD: selective digestive decontamination; RRT: renal replacement therapy; UK: United Kingdom

* P-value calculated by ANOVA

| **Table S4. Prior probability estimates* by training** | | | | | | |
| --- | --- | --- | --- | --- | --- | --- |
| **Trial name** | **Mean+/-SD prior probability by training (%)** | | | | **P-value** | |
|  | **Intensive Care Specialist (n=101)** | **Other Specialist (n=21)** | **Training in an area of medicine other than Intensive Care Medicine (n=15)** | **Training to be a Specialist in Intensive Care Medicine (n=29)** | |  |
| A Confirmatory Phase II/III Study Assessing Efficacy, Immunogenicity And Safety Of Ic43 Recombinant Pseudomonas Vaccine In Intensive Care Patients | 10.9  ±15.2 | 18.3  ±21.9 | 15.0  ±17.3 | 10.2  ±16.4 | | 0.23 |
| ADjunctive coRticosteroid trEatment iN criticAlly ilL Patients With Septic Shock | 27.0  ±24.9 | 24.4  ±26.4 | 36.7  ±26.6 | 26.3  ±22.1 | | 0.48 |
| Early Spontaneous Breathing in ARDS | 15.0  ±20.4 | 19.7  ±19.4 | 34.9  ±28.4 | 21.3  ±29.0 | | 0.02 |
| Non-sedation Versus Sedation With a Daily Wake-up Trial in Critically Ill Patients Receiving Mechanical Ventilation | 24.9  ±26.3 | 28.0  ±28.7 | 24.9  ±25.0 | 28.6  ±25.7 | | 0.90 |
| Stress Ulcer Prophylaxis in the Intensive Care Unit | 15.9  ±22.1 | 14.0  ±18.2 | 24.2  ±30.9 | 24.1  ±25.0 | | 0.22 |
| The Augmented Versus Routine Approach to Giving Energy Trial | 18.3  ±20.5 | 26.4  ±23.6 | 34.9  ±27.5 | 19.1  ±23.5 | | 0.04 |
| The SuDDICU Cluster RCT of Antibiotic Prophylaxis in Critical Illness | 35.9  ±29.2 | 30.8  ±32.2 | 30.1  ±24.7 | 37.0  ±28.4 | | 0.78 |
| Ticagrelor in Severe CAP | 10.0  ±19.0 | 13.4  ±22.7 | 18.5  ±28.2 | 7.6  ±11.8 | | 0.31 |
| Tranexamic acid for the treatment of gastrointestinal haemorrhage: an international randomised, double blind placebo controlled trial | 31.8  ±26.1 | 49.6  ±30.8 | 59.0  ±27.6 | 42.7  ±23.8 | | <0.01 |
| Tranexamic Acid for the treatment of significant traumatic brain injury: an international, randomised, double blind, placebo controlled trial | 26.3  ±24.6 | 32.4  ±27.3 | 37.9  ±30.1 | 27.9  ±23.6 | | 0.34 |

Abbreviations: APRV: airway pressure release ventilation; ARDS: acute respiratory distress syndrome; CAP: Community Acquired Pneumonia; GI: gastrointestinal; ICU: Intensive Care Unit; IQR: interquartile range; SD: standard deviation; SDD: selective digestive decontamination; RRT: renal replacement therapy

* data required to perform the modified sample size calculation for this cluster trial could not be derived from the survey response data.

| **Table S5. Largest effect size considered plausible by training** | | | | | | |
| --- | --- | --- | --- | --- | --- | --- |
| **Trial name** | **Mean+/-SD prior probability by training (%)** | | | | **P-value** | |
|  | **Intensive Care Specialist (n=101)** | **Other Specialist (n=21)** | **Training in an area of medicine other than Intensive Care Medicine (n=15)** | **Training to be a Specialist in Intensive Care Medicine (n=29)** | |  |
| A Confirmatory Phase II/III Study Assessing Efficacy, Immunogenicity And Safety Of Ic43 Recombinant Pseudomonas Vaccine In Intensive Care Patients | 6.8  ±7.7 | 8.8  ±11.9 | 5.5  ±4.9 | 5.6  ±5.3 | | 0.59 |
| ADjunctive coRticosteroid trEatment iN criticAlly ilL Patients With Septic Shock | 8.8  ±8.3 | 11.2  ±22.8 | 12.2  ±15.8 | 8.1  ±6.9 | | 0.63 |
| Early Spontaneous Breathing in ARDS | 7.3  ±8.6 | 8.8  ±11.7 | 15.1  ±27.1 | 8.1  ±8.1 | | 0.19 |
| Non-sedation Versus Sedation With a Daily Wake-up Trial in Critically Ill Patients Receiving Mechanical Ventilation | 10.1  ±10.6 | 12.5  ±13.9 | 5.4  ±3.3 | 12.6  ±11.7 | | 0.26 |
| Stress Ulcer Prophylaxis in the Intensive Care Unit | 5.4  ±7.8 | 6.3  ±8.4 | 4.4  ±4.1 | 5.9  ±5.5 | | 0.90 |
| The Augmented Versus Routine Approach to Giving Energy Trial | 5.6  ±7.0 | 6.9  ±11.5 | 9.2  ±14.0 | 4.4  ±5.8 | | 0.35 |
| The SuDDICU Cluster RCT of Antibiotic Prophylaxis in Critical Illness | 6.5  ±5.9 | 6.9  ±6.9 | 3.6  ±2.0 | 7.1  ±4.6 | | 0.32 |
| Ticagrelor in Severe CAP | 6.4  ±8.5 | 8.4  ±11.1 | 6.8  ±9.5 | 4.4  ±6.6 | | 0.55 |
| Tranexamic acid for the treatment of gastrointestinal haemorrhage: an international randomised, double blind placebo controlled trial | 5.0  ±9.2 | 4.2  ±2.5 | 6.1  ±6.0 | 4.1  ±2.1 | | 0.87 |
| Tranexamic Acid for the treatment of significant traumatic brain injury: an international, randomised, double blind, placebo controlled trial | 5.2  ±5.8 | 5.9  ±5.6 | 7.9  ±8.9 | 4.6  ±4.0 | | 0.40 |

Abbreviations: APRV: airway pressure release ventilation; ARDS: acute respiratory distress syndrome; CAP: Community Acquired Pneumonia; GI: gastrointestinal; ICU: Intensive Care Unit; IQR: interquartile range; SD: standard deviation; SDD: selective digestive decontamination; RRT: renal replacement therapy

* data required to perform the modified sample size calculation for this cluster trial could not be derived from the survey response data.

| **Figure S1: Frequency of estimates of prior probability for each trial** | | | | | | |
| --- | --- | --- | --- | --- | --- | --- |
|  |  |  |  |  |  |  |
|  | 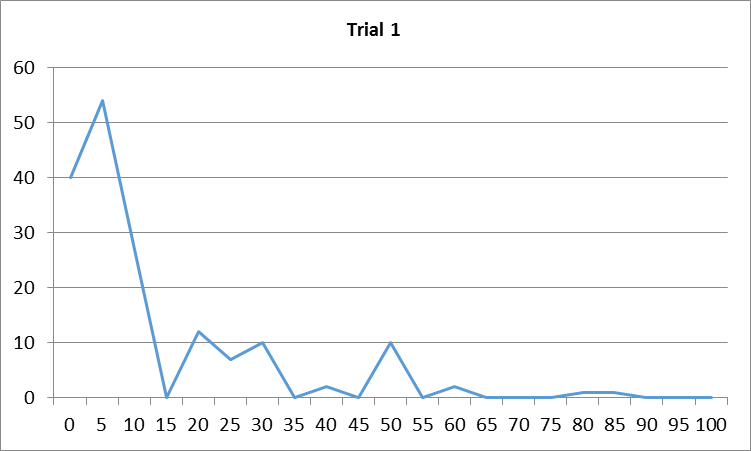 | 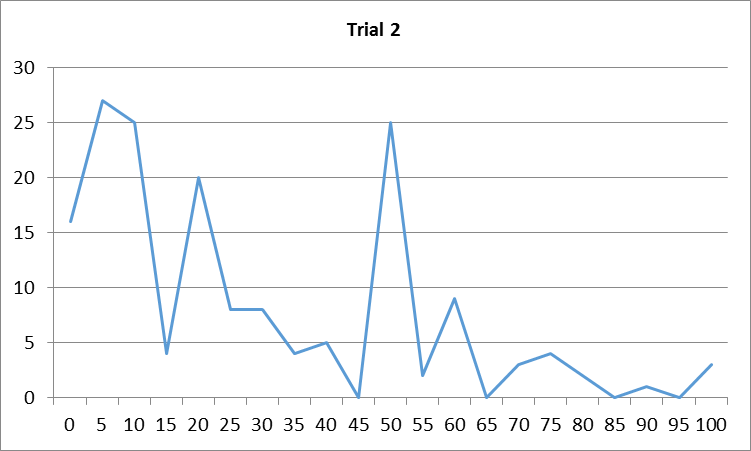 | 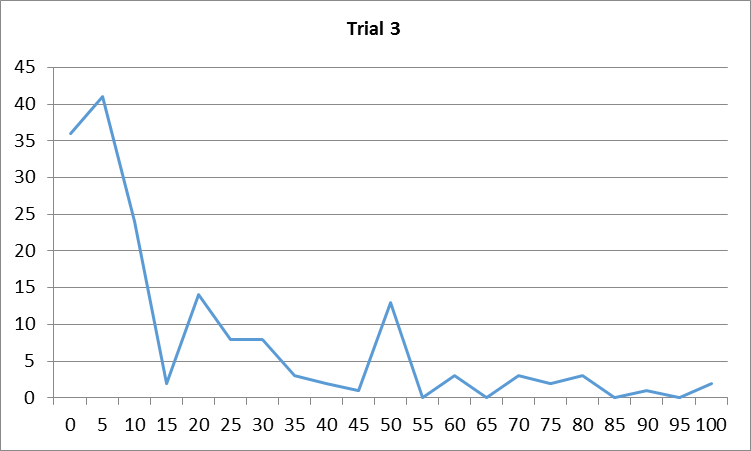 | 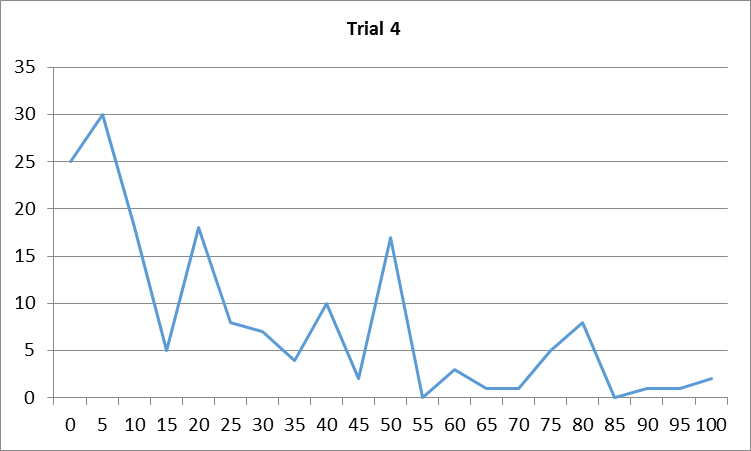 | 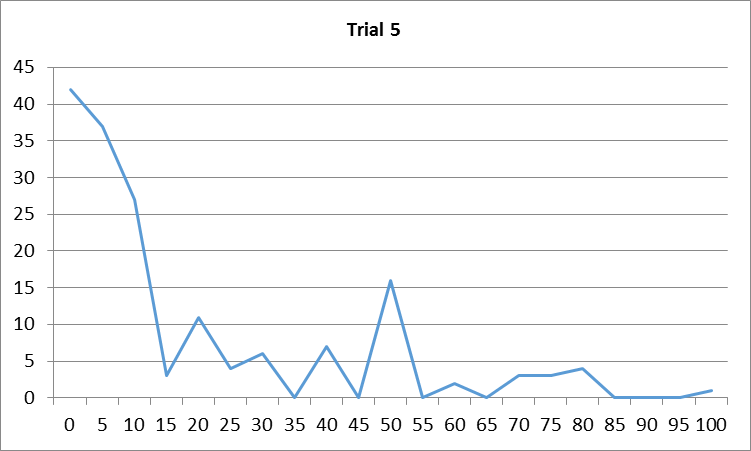 |  |
|  | 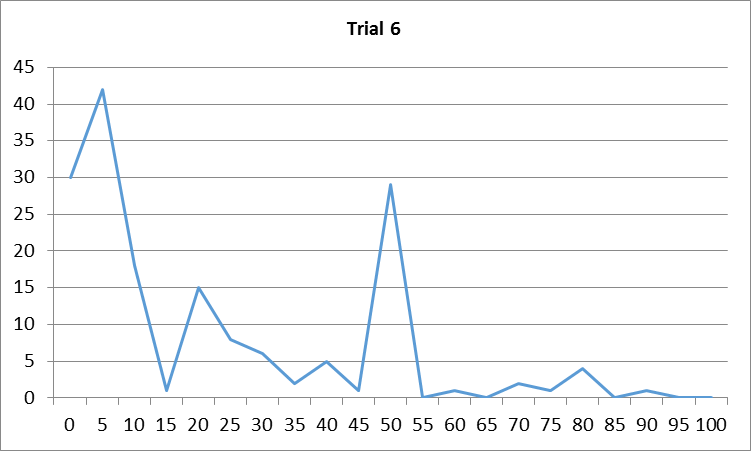 | 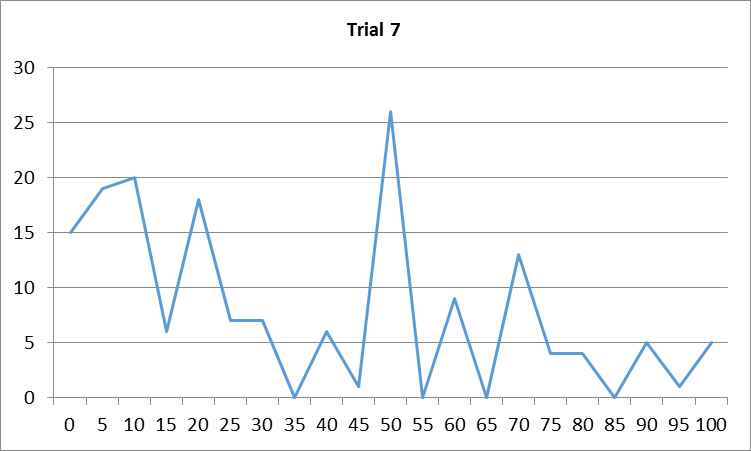 | 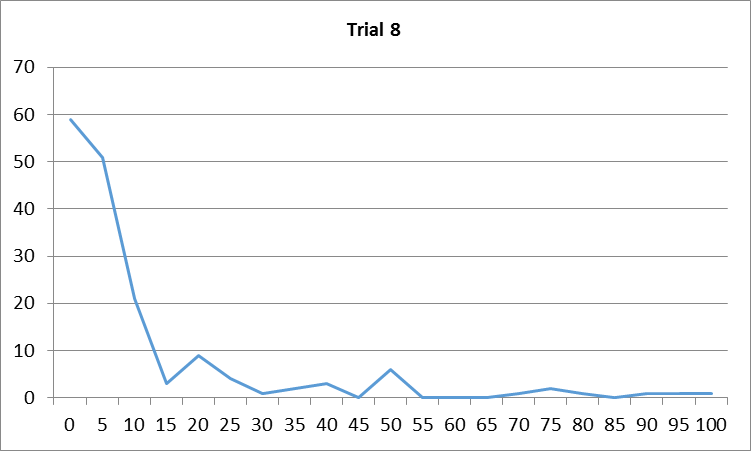 | 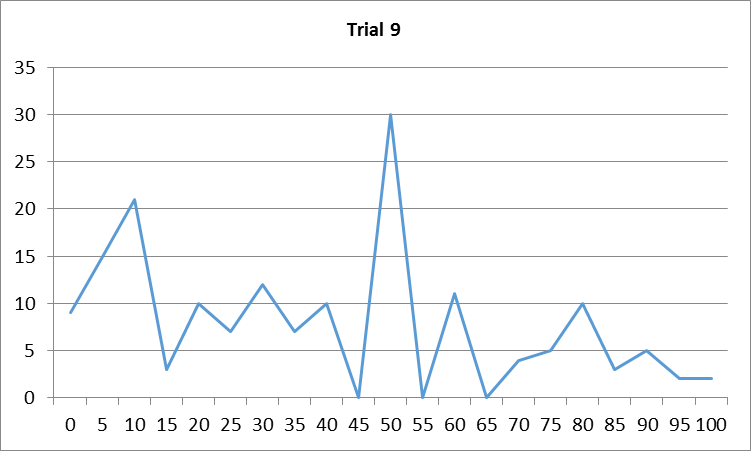 | 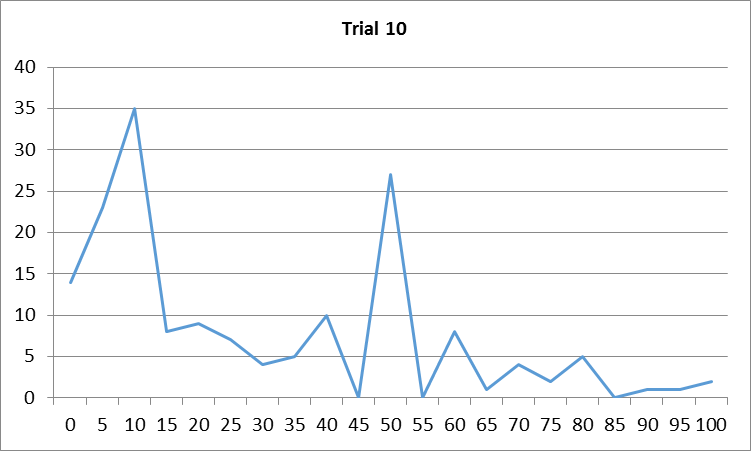 |  |
|  |  |  |  |  |  |  |
| Vertical axes show the percentage of respondents who chose each estimate and the horizontal axes show the probability estimates expressed as a percentage  Trial 1: A Confirmatory Phase II/III Study Assessing Efficacy, Immunogenicity And Safety Of Ic43 Recombinant Pseudomonas Vaccine In Intensive Care Patients  Trial 2: ADjunctive coRticosteroid trEatment iN criticAlly ilL Patients With Septic Shock Trial 3: Early Spontaneous Breathing in ARDS  Trial 4: Non-sedation Versus Sedation With a Daily Wake-up Trial in Critically Ill Patients Receiving Mechanical Ventilation  Trial 5: Stress Ulcer Prophylaxis in the Intensive Care Unit  Trial 6: The Augmented Versus Routine Approach to Giving Energy Trial  Trial 7: The SuDDICU Cluster RCT of Antibiotic Prophylaxis in Critical Illness  Trial 8: Ticagrelor in Severe CAP  Trial 9: Tranexamic acid for the treatment of gastrointestinal haemorrhage: an international randomised, double blind placebo controlled trial  Trial 10: Tranexamic Acid for the treatment of significant traumatic brain injury: an international, randomised, double blind, placebo controlled trial | | | | | | |
| **Figure S2: Frequency of estimates of the largest absolute mortality reduction plausible for each trial** | | | | | | |
|  |  |  |  |  |  |  |
|  | 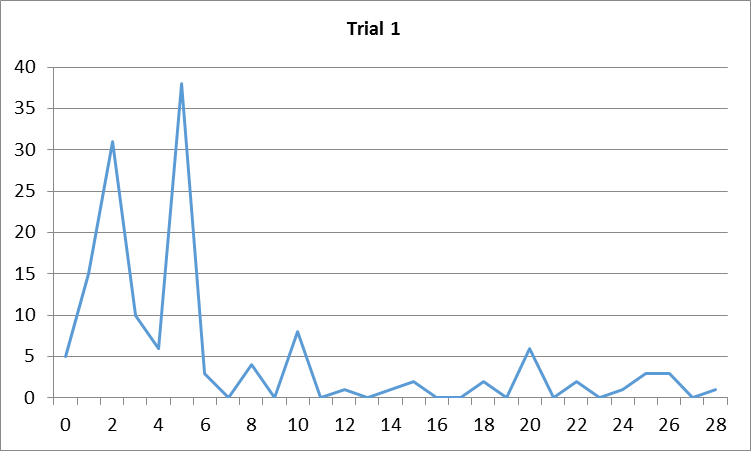 | 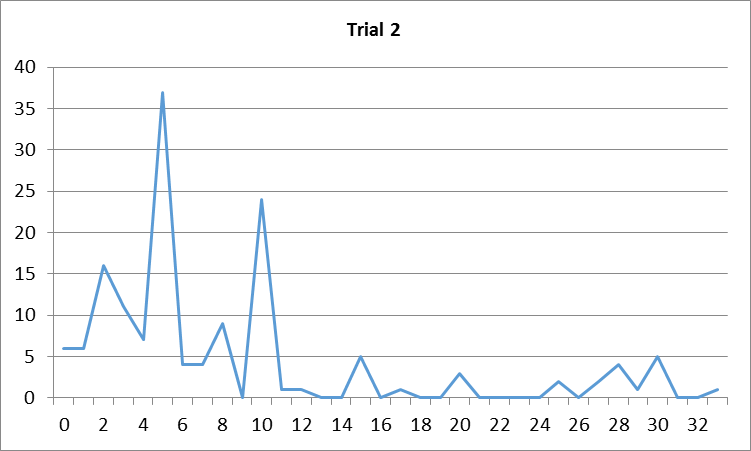 | 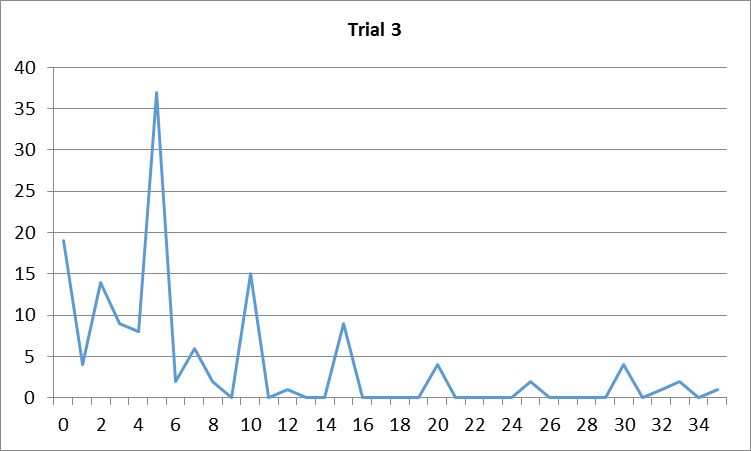 | 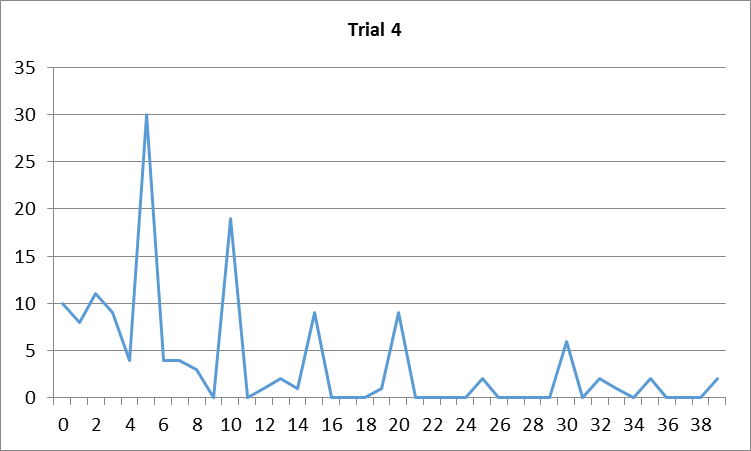 | 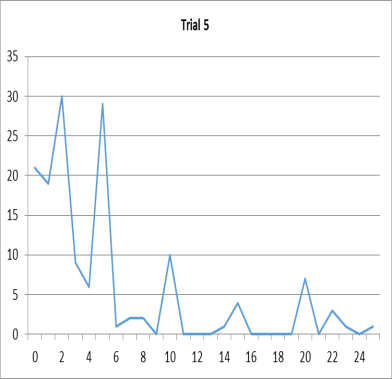 |  |
|  | 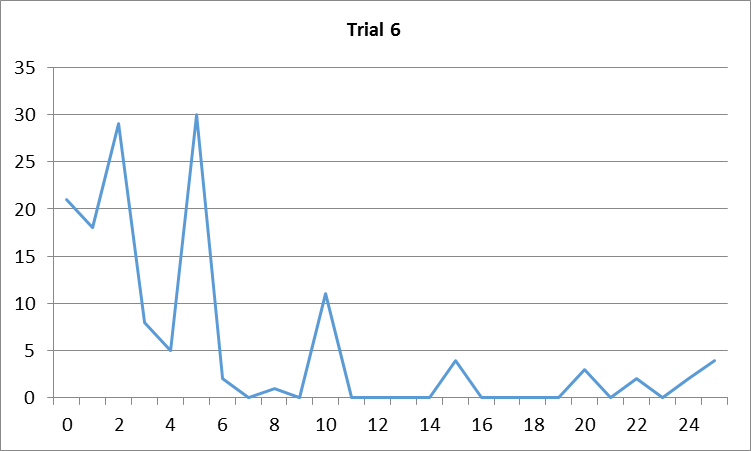 | 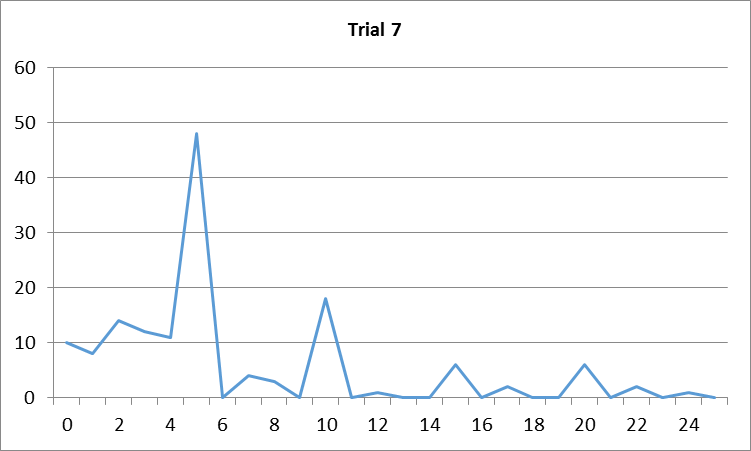 | 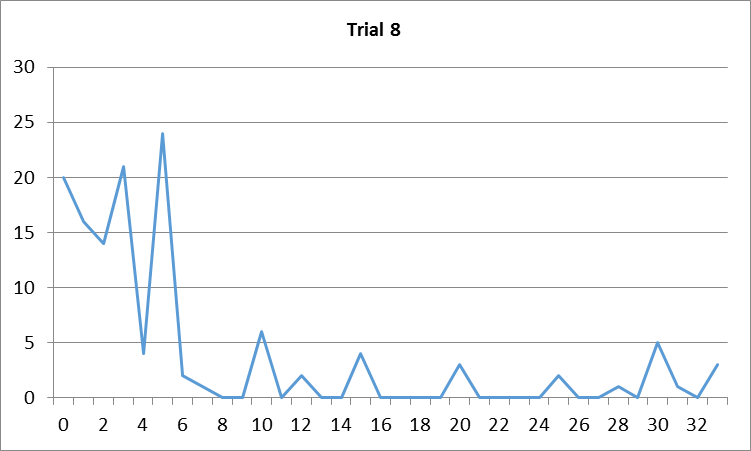 | 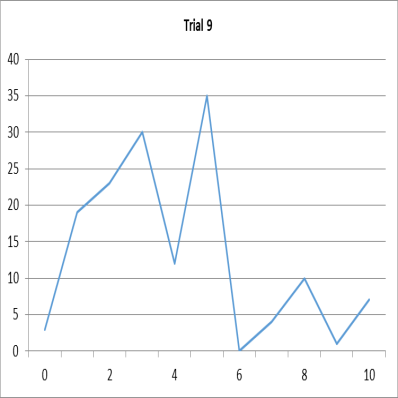 | 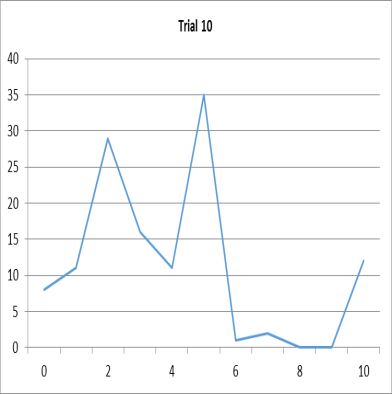 |  |
|  |  |  |  |  |  |  |
| Vertical axes show the percentage of respondents who chose each absolute reduction in mortality and the horizontal axes show the largest absolute reduction in mortality considered plausible  Trial 1: A Confirmatory Phase II/III Study Assessing Efficacy, Immunogenicity And Safety Of Ic43 Recombinant Pseudomonas Vaccine In Intensive Care Patients  Trial 2: ADjunctive coRticosteroid trEatment iN criticAlly ilL Patients With Septic Shock Trial 3: Early Spontaneous Breathing in ARDS  Trial 4: Non-sedation Versus Sedation With a Daily Wake-up Trial in Critically Ill Patients Receiving Mechanical Ventilation  Trial 5: Stress Ulcer Prophylaxis in the Intensive Care Unit  Trial 6: The Augmented Versus Routine Approach to Giving Energy Trial  Trial 7: The SuDDICU Cluster RCT of Antibiotic Prophylaxis in Critical Illness  Trial 8: Ticagrelor in Severe CAP  Trial 9: Tranexamic acid for the treatment of gastrointestinal haemorrhage: an international randomised, double blind placebo controlled trial  Trial 10: Tranexamic Acid for the treatment of significant traumatic brain injury: an international, randomised, double blind, placebo controlled trial | | | | | | |
